# Zebrafish models in glioma research: advances in methodologies, mechanistic insights, and therapeutic frontiers

**DOI:** 10.3389/fimmu.2025.1601656

**Published:** 2025-06-24

**Authors:** Runchao Tao, Junying Qu, Jing Zhao, Baihui Wu, Huaibiao Xu, Liangwei Yang, Hongru Qin, Rongbing Chen, Qinsi Yang, Yongwei Cheng, Wei Wu, Da Sun, Min Cao

**Affiliations:** ^1^ Institute of Life Sciences and Biomedical Collaborative Innovation Center of Zhejiang Province, Wenzhou University, Wenzhou, China; ^2^ The First School of Medicine, School of Information and Engineering, Wenzhou Medical University, Wenzhou, China; ^3^ Department of Biomedical Engineering, City University of Hong Kong, Kowloon, Hong Kong SAR, China; ^4^ Wenzhou Institute, University of Chinese Academy of Sciences, Wenzhou, China; ^5^ National Engineering Research Center of Cell Growth Factor Drugs and Protein Biologics, Wenzhou Medical University, Wenzhou, China; ^6^ Key Laboratory for Biorheological Science and Technology of Ministry of Education, State and Local Joint Engineering Laboratory for Vascular Implants, Bioengineering College of Chongqing University, Chongqing, China; ^7^ JinFeng Laboratory, Chongqing, China; ^8^ The Quzhou Affiliated Hospital of Wenzhou Medical University, Quzhou People’s Hospital, Quzhou, China

**Keywords:** glioma, zebrafish, tumor microenvironment, xenografting, gene editing

## Abstract

Glioma is the most common primary malignant brain tumor, which faces great challenges in clinical treatment due to its high invasiveness and resistance to existing treatments. In recent years, the zebrafish model has gradually become an important tool for glioma research due to its advantages such as easy genetic manipulation, strong optical transparency, and suitability for high-throughput imaging and drug screening. This article systematically reviews the three main strategies for zebrafish glioma modeling - chemical mutagenesis, genetic engineering and xenotransplantation, and describes their research applications in tumorigenesis, invasion process and treatment response. At the same time, this article deeply analyzes the limitations of the zebrafish model in terms of temperature differences, delayed development of the blood-brain barrier and immature immune system, and introduces the cutting-edge progress in recent years in the fields of CRISPR-mediated immune regulation, construction of high-temperature resistant strains and development of humanized models. Through a comprehensive review of current research applications, key challenges and future development directions, this article emphasizes the potential value of the zebrafish model as an important supplement to the mammalian model in exploring the immune mechanism of glioma and developing innovative treatment strategies.

## 1 Introduction

Gliomas are the most prevalent primary intracranial tumor, originating from glial cells (1, 2) and accounting for nearly 80% of all malignant brain tumors. (3, 4) These tumors are characterized by their infiltrative growth pattern, which not only complicates surgical resection, but also contributes significantly to the high recurrence rates observed in affected patients. (5, 6) Glioblastoma multiforme (GBM), (7) the most aggressive and lethal subtype of glioma, has a global incidence of 0.59 to 3.69 per 100,000 individuals, with the highest incidence observed in individuals aged 75–84. (8) According to the WHO classification, adult diffuse gliomas are simplified into the following three types: Astrocytoma, Oligodendroglioma, Glioblastoma. (9) Despite advances in treatment, the prognosis for GBM remains poor, with a median survival of 12–18 months post-diagnosis and a 5-year survival rate of less than 10%. (10) This underscores the urgent need for novel therapeutic approaches and better understanding of the molecular mechanisms underlying glioma pathogenesis.

The pathogenesis of glioma remains incompletely understood. Established risk factors include exposure to ionizing radiation, high-penetrance genetic mutations, and hereditary syndromes, (11) while environmental factors like nitrite-rich diets and potential viral or bacterial infections have been implicated as secondary contributors. (12) Current treatments, including surgical resection, temozolomide chemotherapy, and radiation therapy, show limited efficacy, (13) as nearly all gliomas recur within six months of treatment. This recurrence is largely due to the invasive nature of gliomas, which infiltrate adjacent brain tissue, making complete resection difficult and leading to post-surgical relapse. (14, 15).

Given these challenges, the development of robust and clinically translatable glioma models is essential. Traditional animal models, including genetically engineered mice (GEM), immunocompromised mice used for xenotransplantation, and large animals like dogs and pigs, provide valuable insights but also come with inherent limitations. These include high costs, ethical concerns, and a limited ability to replicate the human glioma microenvironment accurately, which hampers their translational potential for drug screening and therapeutic development.

However, these models often fail to fully replicate the intricate interactions within the tumor microenvironment (TME), which are essential for understanding glioma biology and evaluating new therapies (16). **Table 1** provides an overview of commonly used glioma animal models, highlighting their respective advantages and limitations.

**Table 1:** Common glioma animal models.

Animals model	Type	Modeling method	Advantages	Limitations	References
Immunocompromised mice	Xenotransplantation	Injection of human glioma cells into mouse brains	Short modeling time; High efficiency	Lacks functional immune system; Limited TME	(17)
C57BL/6, BALBc, FVB/N mice	Isogenic model	Tumor induction via carcinogens or genetic modifications	High modeling efficiency; species-specific responses	Limited to murine tissues; May not fully represent human glioma	(18)
GEM mice	Genetic engineering	Tumor formation through gene manipulation	Low mortality; Precise genetic control	High cost; Lengthy experimental timelines	(19)
Electroporation mice model	Electroporation	Plasmids carrying oncogenes are introduced into brain progenitor cells by *in utero* electroporation	Rapid induction of tumor formation is possible, making it suitable for studying tumor heterogeneity and cell origin.	Traditional plasmids are easily diluted or inactivated during cell division, resulting in unstable gene expression	(20)
Transposon-mediated genetic mosaic model in mice	Transposon-mediated	Tumor formation can be induced by co-electroporation of plasmids, and cell populations with different genetic alterations can be tracked by fluorescent labeling	Modeling genetic heterogeneity within tumors, suitable for studying tumor progression and cell-cell interactions	Technically complex operation	(21)
Landrace, Yucatan minipigs	Xenotransplantation	Transplantation of human glioma cell under immunosuppression	Scalable large-animal model; robust tumor environment	High cost; Limited data on porcine immune-tumor interactions	(22)
Göttingen mini pig	Genetic engineering	Injection of lentiviral vectors expressing platelet-derived growth factor-β, constitutively active HRAS, and shRNA-p53	Modeling is fast and solves the problem of slow growth of large mammal modeling.	Whether tumors progress due to clonal expansion of virus-infected cells or through recruitment and transformation of resident glial progenitor cells is unclear	(23)
Brachycephalic dogs	Naturally occurring	Spontaneous glioma formation	Close resemblance to human glioma	Limited sample size; ethical and practical concerns	(24)
F344/IcoCrl rat	Xenotransplantation	Seed 20,000 F98 cells in 5 μL of phosphate-buffered saline (PBS) in the right entorhinal cortex (GB1-15).	High modeling efficiency	Video EEG monitoring of a limited number of animals	(25)
New Zealand rabbit	Microinjection	Introduce the microneedle (gauge 26) into the brain and inject GBM1 cells at the coordinates	Modeling has a higher survival rate	Requires additional immunosuppressive medications	(26)
Rat	Microinjection	Injection of PDGF-B, HRAS-G12V and shRNA-p53 virus mixture into rats	Use of lentivirus enhances the translational properties of PDGF-B in spinal cord glial cells, resulting in a more penetrant disease model	lower survival rate	(27)
Macaque	Radiation induction	Using radiation to induce glioma in macaques	Close to the way humans induce glioma	No common glioblastoma biomarker	(28)
Fruit fly	Microinjection	dEGFRl and dp110CAAX mutant glial cells injected into host Drosophila abdomen	High modeling efficiency	The tumor will irritate the fly’s trachea or occupy the existing trachea or oxygen-carrying tubules, causing the fly’s death.	(29)

Zebrafish (*Danio rerio*) models have emerged as promising alternatives for glioma research. They offer several advantages over traditional models, including genetic homology with humans (approximately 87%), optical transparency during early development, and a cost-effective platform for large-scale studies. (30) The transparency of zebrafish embryos and larvae allow real-time imaging of tumor growth, angiogenesis, and cell migration at the single-cell level. (31) Furthermore, the lack of an adaptive immune system in zebrafish until around 28 days post-fertilization allows for the direct observation of glioma progression without immune interference. (32) These advantages make zebrafish models particularly useful for high-throughput drug screening and the investigation of glioma-associated genetic mechanisms. (33) Genetic tools, such as CRISPR-Cas9, facilitate precise manipulation of glioma-related genes like *TP53*, *NF1*, and *RB1*, enabling the study of gene-environment interactions and the impact of potential carcinogens. (34) Moreover, zebrafish models have been successfully used to test targeted therapies, providing a unique opportunity to examine the effects of treatment at a cellular level *in vivo*. (35) The use of chemical mutagenesis, genetic engineering, and xenotransplantation further enhances the utility of zebrafish models for studying glioma pathogenesis and testing new therapeutic strategies. (36) We compared the zebrafish and mouse glioma models in **Table 2**.

**Table 2:** Comparison of glioma models established in mice and zebrafish.

Animals	Modeling method	Advantage	Modeling cost	Immune response	References
Mouse	Allograft models, xenograft models, transgenic models, virally mediated	With a complete innate and adaptive immune system, it is an ideal model for studying the tumor immune microenvironment and immunotherapy response	High cost	They have a complete innate and immune system soon after birth, so they are more suitable for studying tumor immune activity and immunotherapy mechanisms. More suitable for studying tumor immune dynamics and immunotherapy mechanisms	(37)
Zebrafish	Xenograft model, chemical-induced model, genetically engineered model	Low cost, fast reproduction, and suitable for high-throughput screening, so it has irreplaceable value in early mechanism exploration and drug screening, and is suitable for dynamic observation of tumor cell migration, invasion and angiogenesis	Cheap	The innate immune system is fully developed by day 2 after fertilization, including macrophages and neutrophils, but the adaptive immune system (T cells and B cells) usually does not mature until week 3–4. Zebrafish models are more suitable for studying early tumor growth, angiogenesis and innate immune response	(38)

This review aim to examine the structural and genetic features of the zebrafish brain, the mechanisms underlying glioma pathogenesis, and current modeling techniques, including chemical mutagenesis, genetic engineering, and xenotransplantation, to construct glioma models in zebrafish. Further, we address the limitations and prospective advancements of the zebrafish model, positioning it as a valuable resource for high-throughput drug screening and as a scalable model for translational glioma research.

## 2 Growth sites of glioma in zebrafish and related regulatory genes

Zebrafish, a widely used vertebrate model organism, offers significant advantages for glioma research due to their genetic similarity to humans, particularly in neuronal and glial cell types. Genes involved in gliomagenesis, including Gfap, Pcna, p*Akt*, Snail, Nestin, and cyclin D1, exhibit high conservation between species, thus supporting the use of zebrafish to investigate human glioma’s molecular mechanisms. (39) This genetic fidelity makes zebrafish an invaluable platform for understanding glioma biology, uncovering potential therapeutic targets, and exploring treatment responses.

### 2.1 Zebrafish brain structure and its relevance to glioma modeling

The zebrafish brain, a primary site for glioma invasion in experimental models, consists of five principal regions: the telencephalon, diencephalon, midbrain, cerebellum, and medulla oblongata. (40) These regions are homologous to those found in the human brain, making zebrafish an effective model for studying glioma progression and tumor-brain interactions (**Table 3**).

**Table 3:** Comparison of zebrafish and human brain structures in glioma research.

Region	Zebrafish Brain Function	Human Brain Equivalent	Relevance to Glioma Research	References
Telencephalon	Olfactory lobe, cerebral hemispheres	Forebrain	Glioma invasion and proliferation	(41)
Diencephalon	Hypothalamus, epithalamus, thalamus	Diencephalon	Tumor-host brain interaction	(42)
Midbrain	Optic tectum, tegmentum	Midbrain	Visual disturbances in glioma	(43)
Cerebellum	Motor control and coordination	Cerebellum	Glioma impact on motor function	(44)
Medulla Oblongata	Autonomic functions, neural tracts	Medulla Oblongata	Glioma-induced neural dysfunction	(45)

**Figure 1:**
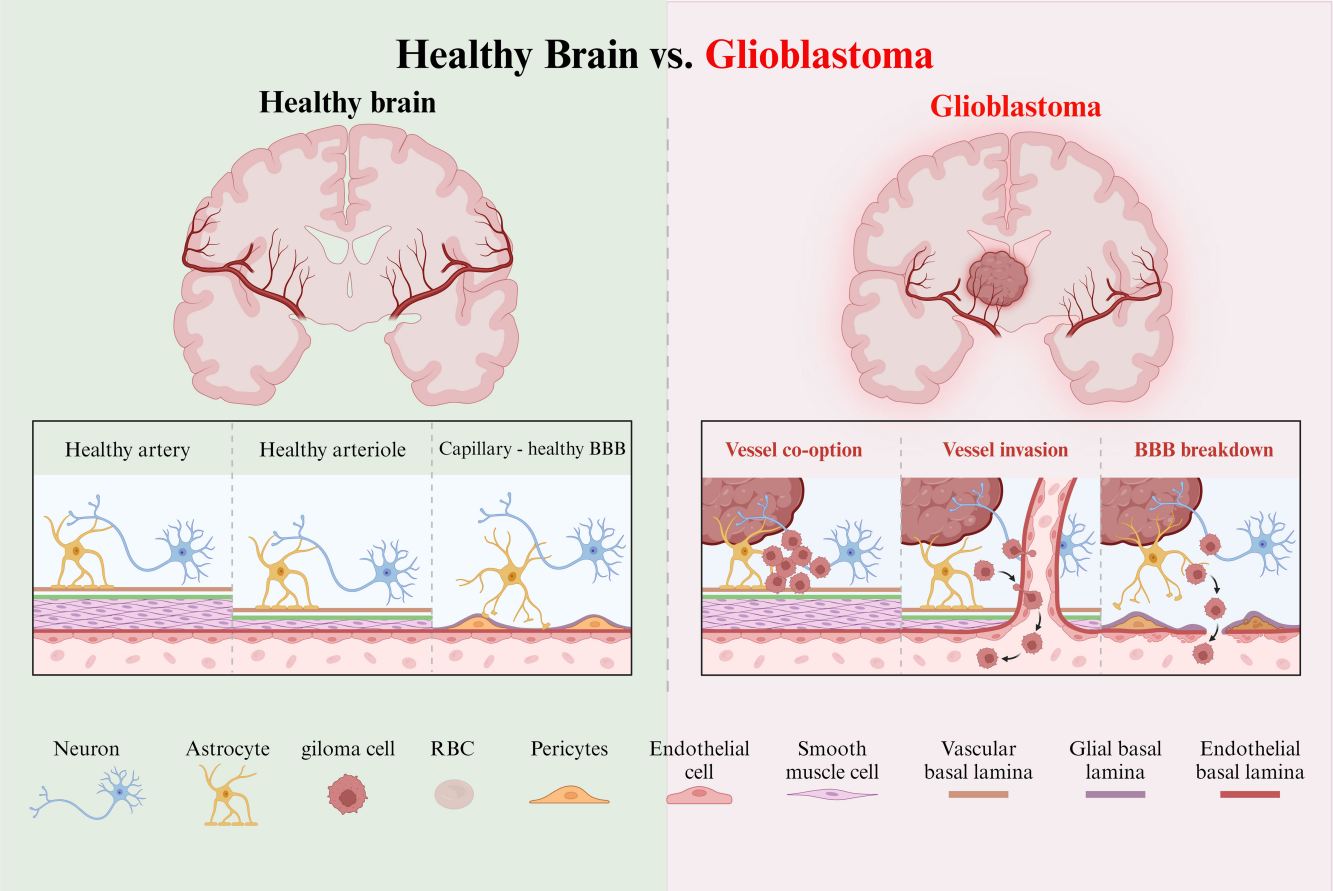
Healthy and tumor brain vascular architecture. Left panel:The perivascular space is demarcated by the vascular basement membrane and the glial basement membrane. Molecules diffuse or transport at the capillary level. Right panel: GBM is a highly angiogenic and infiltrative tumor. Cells invade along blood vessels to support tumor growth. GBM displaces astrocyte endfeet and alters pericyte stability, leading to perivascular niche and cell escape.

1. Telencephalon: Situated at the anterior, this region includes the olfactory lobe, olfactory sac, and cerebral hemispheres. (46) It shares structural features with the human forebrain, such as a large surface area and dense neural connectivity, providing an ideal site for glioma growth. The high density of unmyelinated fibers and glial cells in this region closely mimics the glioma microenvironment seen in humans, allowing for detailed studies of glioma invasion and proliferation (41).
2. Diencephalon: Located posterior to the telencephalon, the diencephalon includes the epithalamus, hypothalamus, thalamus, pretectum, preoptic area, and posterior tubercle. (42) The pineal gland is located on the dorsal side of the diencephalon, with a heart-shaped funnel on the ventral side, and the front end of the funnel is connected to the pituitary gland. The hypothalamus is instrumental in zebrafish neuroendocrine regulation, whereas the epithalamus, containing the pineal gland, serves critical sensory processing functions. (47) The functional similarity of the zebrafish diencephalon to its human counterpart offers insight into how gliomas interact with hormonally regulated brain regions, providing a platform for studying glioma-host brain dynamics.
3. Midbrain: The midbrain is located behind the diencephalon and includes the optic tectum, tegmentum and carina Neuronal pathways from retinal ganglion cells form the optic nerve, transmitting visual data to the tectum. (48) Although structurally simpler than the human optic pathway, the zebrafish midbrain’s neural connectivity allows for the investigation of glioma-induced visual disturbances and glioma-cell interactions within the optic pathway.
4. Cerebellum: The cerebellum is relatively large and integral for zebrafish motor control, composed of a ventral ridge and cerebellar valve, coordinating active movement patterns typical of the species. (49) Its intricate neural circuitry mirrors the complex brain regions affected by glioma, making it a valuable region for studying glioma-induced motor dysfunction and its interaction with tumor expansion.
5. Medulla Oblongata: The medulla is integral for autonomic functions, connecting to the cerebellum through the central ear-shaped process. Its ventricles and neural tracts provide additional avenues for glioma invasion studies. Moreover, zebrafish share a blood-brain barrier (BBB) with mammals, featuring tight junctions and an active transport system that regulates molecular access to brain tissues. (45) This BBB model enables the study of glioma cell infiltration and the evaluation of therapeutic agents that target the BBB (**Figure 1**).

### 2.2 Glioma invasion process in the brain

Glioblastoma is characterized by its heterogeneous cell populations, which originate from glioma stem cells (GSCs) within a vascular niche. GSCs, predominantly located in the subventricular zone, possess stem-like neural properties that facilitate tumor initiation and resistance to therapy (50). These cells play a pivotal role in glioma invasion by exploiting blood vessels in the TME, promoting angiogenesis, and generating a vascular network that supports tumor growth.

Vascular endothelial growth factor (VEGF) signaling drives this angiogenesis, allowing glioma cells to migrate along the vascular basement membrane and bypass immune surveillance. (51) This invasive process leads to glioma cells breaching the BBB, disrupting the glial-vascular interface and facilitating widespread infiltration throughout the brain. As gliomas invade, they induce necrosis in the surrounding brain tissue, further complicating treatment.

Molecular alterations in gliomas are key drivers of their invasive behavior. The Cancer Genome Atlas has identified several dysregulated pathways, including the RTK/Ras/PI3K, p53, and retinoblastoma pathways, which contribute to glioma progression and resistance to therapeutic strategies. (52) These alterations drive aggressive proliferation, migration, and evasion of cell death, which underpins glioblastoma’s aggressive nature and poor prognosis.

### 2.3 Glioma-related regulatory genes and pathways

**Figure 2:**
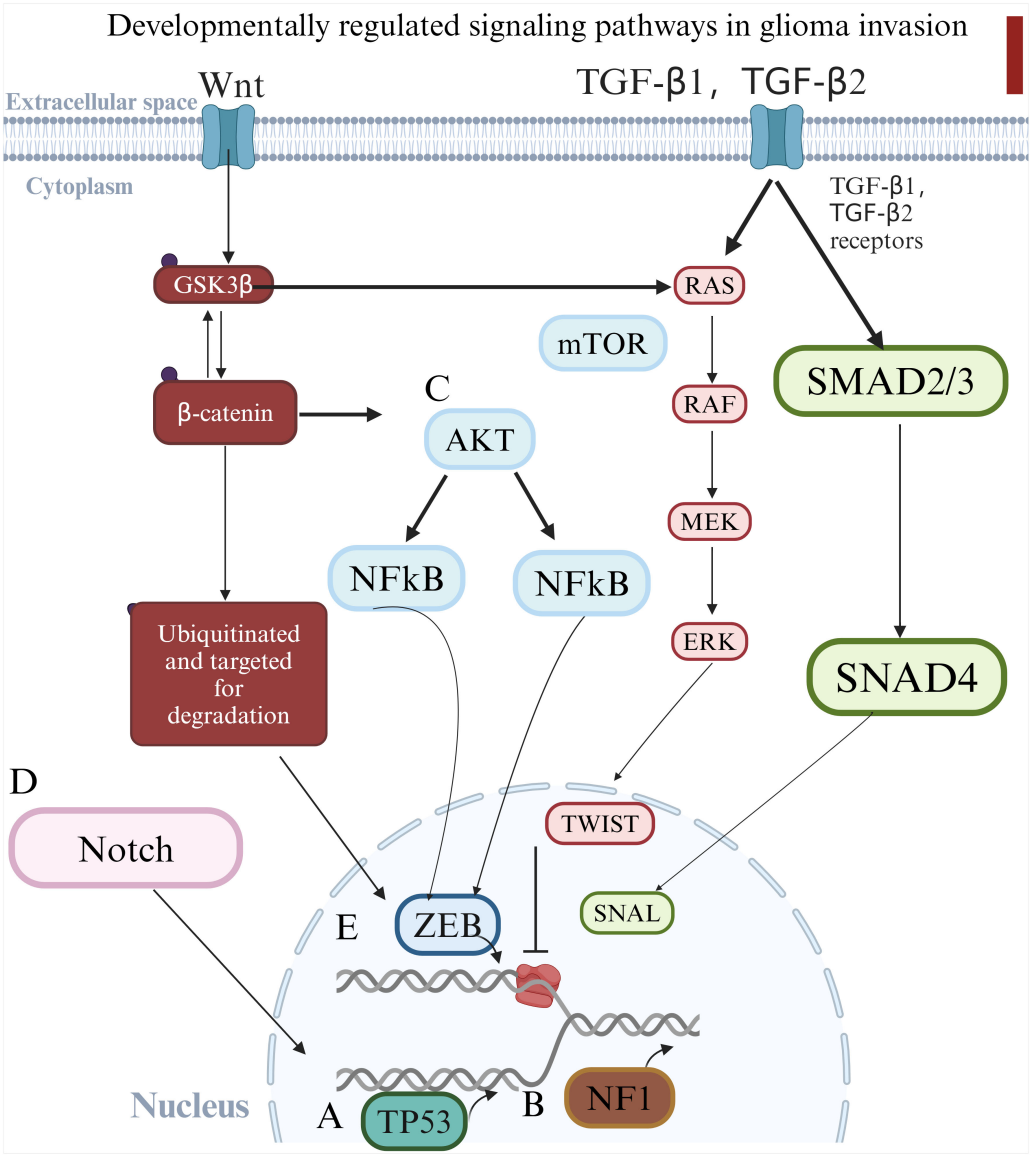
Schematic diagram illustrating the key genetic alterations and signaling pathways involved in glioma progression. **(A)** *TP53* mutations lead to apoptosis resistance; **(B)** *NF1* mutations drive mesenchymal transformation; **(C)** AKT activation supports cell survival and migration; **(D)** Dysregulated Notch signaling enhances glioma stemness and invasiveness. These pathways collectively contribute to glioma aggressiveness and therapeutic resistance. **(E)** Causes epithelial-mesenchymal transition and accelerates glioma invasion.

Glioma progression is driven by a series of genetic alterations that disrupt cellular growth regulation, inactivate tumor suppressor genes, and activate survival pathways (**Supplementary Table S1**). (53) Key regulatory genes and pathways implicated in glioma development include *TP53* mutations, loss of *NF1* function, dysregulation of *AKT*, Notch signaling and Wnt pathway and TGF-β pathway (**Figure 2**).

#### 2.3.1 *TP53* mutation and glioma progression

*TP53* mutations represent one of the most frequent genetic alterations in gliomas and are crucial in the progression of these tumors. (54) The *TP53* gene, located on chromosome 17p, encodes the tumor suppressor protein p53, which is pivotal in regulating cell cycle arrest, mediating DNA repair, and initiating apoptosis in response to cellular stress. (55) In gliomas, inactivating mutations in *TP53* lead to the loss of p53 function, resulting in tumorigenic features such as resistance to apoptosis, uncontrolled cell proliferation, and genomic instability. (56) These mutations are associated with poor prognosis, as glioma cells lacking functional p53 exhibit enhanced proliferative capacities and increased resistance to therapies like radiation and chemotherapy. The loss of *p53* not only contributes to tumor initiation but also facilitates glioma recurrence, highlighting the importance of TP53 mutations in glioma pathophysiology.

#### 2.3.2 Neurofibromin 1 mutations and mesenchymal transformation

*Neurofibromin 1* (*NF1)*, a well-known tumor suppressor gene, is frequently mutated in gliomas, particularly in the mesenchymal subtype. (57) *NF1* regulates the RTK/Ras/PI3K signaling pathway, and mutations in this gene lead to loss of function, which promotes cellular transformation and drives the transition of glioma cells towards a more aggressive, mesenchymal phenotype. (58) These mutations increase the motility, invasiveness, and resistance of glioma cells, contributing to a more aggressive tumor progression. Furthermore, *NF1* mutations enhance the proliferation of neural progenitor cells, providing a source of stem-like cells that fuel glioma growth. (59) This shift toward a more invasive and stem-like state underlines the importance of *NF1* mutations in glioma aggressiveness and therapy resistance.

#### 2.3.3 AKT signaling and glioma progression

The AKT signaling pathway, a downstream component of the PI3K signaling cascade, plays a significant role in supporting glioma cell survival, proliferation, and migration. (60) AKT is a serine/threonine kinase that is activated by various growth factors and regulates several downstream targets involved in cellular processes such as survival, growth, metabolism, and angiogenesis. In glioma, aberrant activation of the AKT pathway is commonly observed and is associated with enhanced tumor growth, increased cell migration, and resistance to apoptotic. (61) Hyperactivation of AKT contributes to glioma aggressiveness by supporting tumor cell survival under stressful conditions and facilitating metastasis. Given its pivotal role in glioma biology, AKT remains a promising therapeutic target, with inhibitors currently under investigation in clinical trials to block glioma progression and improve treatment outcomes. (62).

#### 2.3.4 Notch signaling and glioma cell self-renewal

The Notch signaling pathway is integral to glioma development, particularly in regulating GSCs, which contribute to tumor self-renewal, resistance to therapies, and recurrence. (63) Notch signaling is involved in a variety of cellular processes, including differentiation, proliferation, and apoptosis. Dysregulation of Notch signaling, whether through hyperactivation or suppression, is associated with the development and progression of gliomas. (64) Hyperactivation of Notch signaling enhances glioma cell proliferation and invasiveness, while also maintaining the stem-like properties of GSCs. The interaction between Notch1 and the CXCL12/CXCR4 axis has been shown to promote tumor cell invasion and sustain glioma stemness, making this axis a potential therapeutic target. (65) Given its crucial role in maintaining tumor-initiating populations, Notch signaling remains a promising target for glioma therapy.

#### 2.3.5 Wnt pathway and TGF-β pathway

The Wingless/Int1 (Wnt) signaling pathway plays a critical role at different stages of central nervous system (CNS) development and is directly required to regulate self-renewal, proliferation, and differentiation of NPCs in the developing brain. (66) Aberrant activation of the Wnt pathway has been implicated in driving the development and progression of various human cancers.

Another signaling pathway that contributes to GSC invasiveness is the TGF-β pathway. TGF-β signaling plays a critical role in regulating many cellular processes during embryogenesis, cell proliferation, migration, and tissue homeostasis. (67) Although the TGF-β pathway is best known for its tumor suppressor function in epithelial tissues, it also serves as a promoter of tumorigenesis in various solid cancers, including GBM, due to its ability to enhance cell migration and thus cell invasion agent.

Wnt pathway and TGF-β pathway can lead to epithelial-mesenchymal transition(EMT), ultimately leading to loss of epithelial tissue and acquisition of a mesenchymal phenotype.

#### 2.3.6 EGFR/MAPK pathway

In gliomas, especially glioblastoma (GBM), the EGFR/MAPK signaling pathway is widely considered to be one of the key axes driving tumor occurrence and progression. EGFR amplification and constitutively active mutations (such as EGFRvIII) can continuously activate the downstream RAS/RAF/MEK/ERK cascade, thereby promoting cell proliferation, inhibiting apoptosis, and enhancing cell invasiveness. Verhaak et al. divided GBM into four subtypes for the first time through large-scale genomic analysis, and pointed out that the Classical subtype is closely related to EGFR amplification and MAPK pathway activation (68), suggesting that abnormalities in this pathway are one of the core characteristics of specific molecular subtypes. In addition, Suvà et al. found that EGFR/MAPK signaling is essential for maintaining the stemness and proliferation potential of glioma stem cells, and its inhibition can induce cell differentiation and reduce the tumor stem cell phenotype (69). Combined with single-cell sequencing technology, Tirosh and his collaborators further revealed that there are complex developmental hierarchies and cell state variations within gliomas, and that the activity of the EGFR/MAPK pathway is highly heterogeneous in different cell subpopulations (70).

## 3 Construction method for zebrafish glioma model

**Figure 3:**
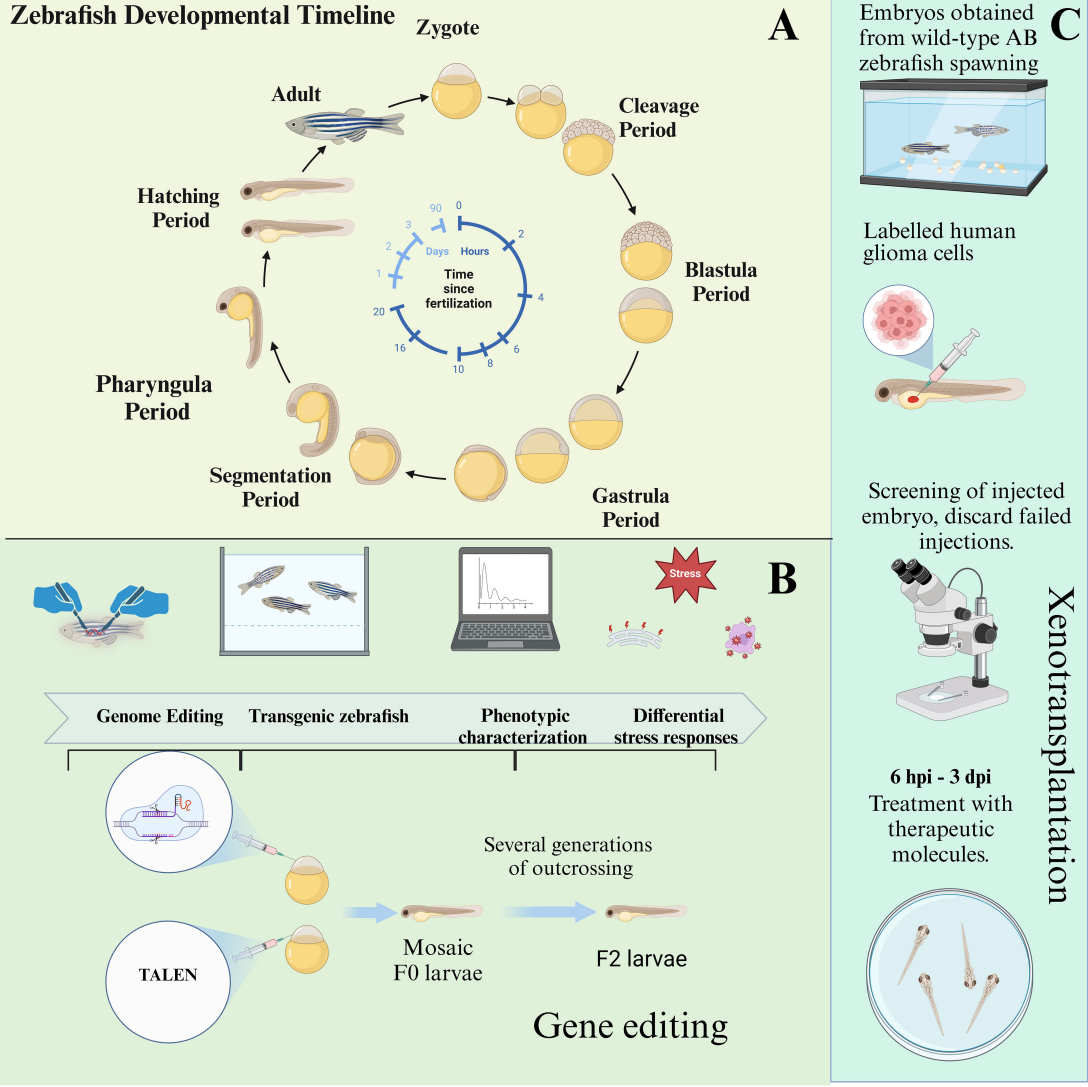
The developmental cycle of zebrafish embryos and the establishment of zebrafish models through genetic engineering and xenotransplantation. **(A)** Depicting the developmental cycle from embryonic to juvenile zebrafish. **(B)** Zebrafish gene editing process. **(C)** Zebrafish xenograft glioma pattern and process.

Developing zebrafish models for glioma research provides critical insights into glioma invasion, development, and cellular migration. Zebrafish embryos, with their rapid development and transparency, enable easy observation of tumor growth and metastasis, making them an efficient model for glioma studies (**Figure 3A**). The following sections summarize the three types of induction methods used to create glioma models in zebrafish (**Figures 3B, C**).

### 3.1 Chemical induction of glioma models

The primary mutagens used to induce glioma in zebrafish are N-Ethyl-N-nitrosourea (ENU) and N-Methyl-N’-nitro-N-nitrosoguanidine (MNNG). These chemicals induce specific mutations in the central nervous system (CNS), particularly the brain, making zebrafish an effective model for studying glioma formation and progress.

#### 3.1.1 ENU-induced glioma model

**Figure 4:**
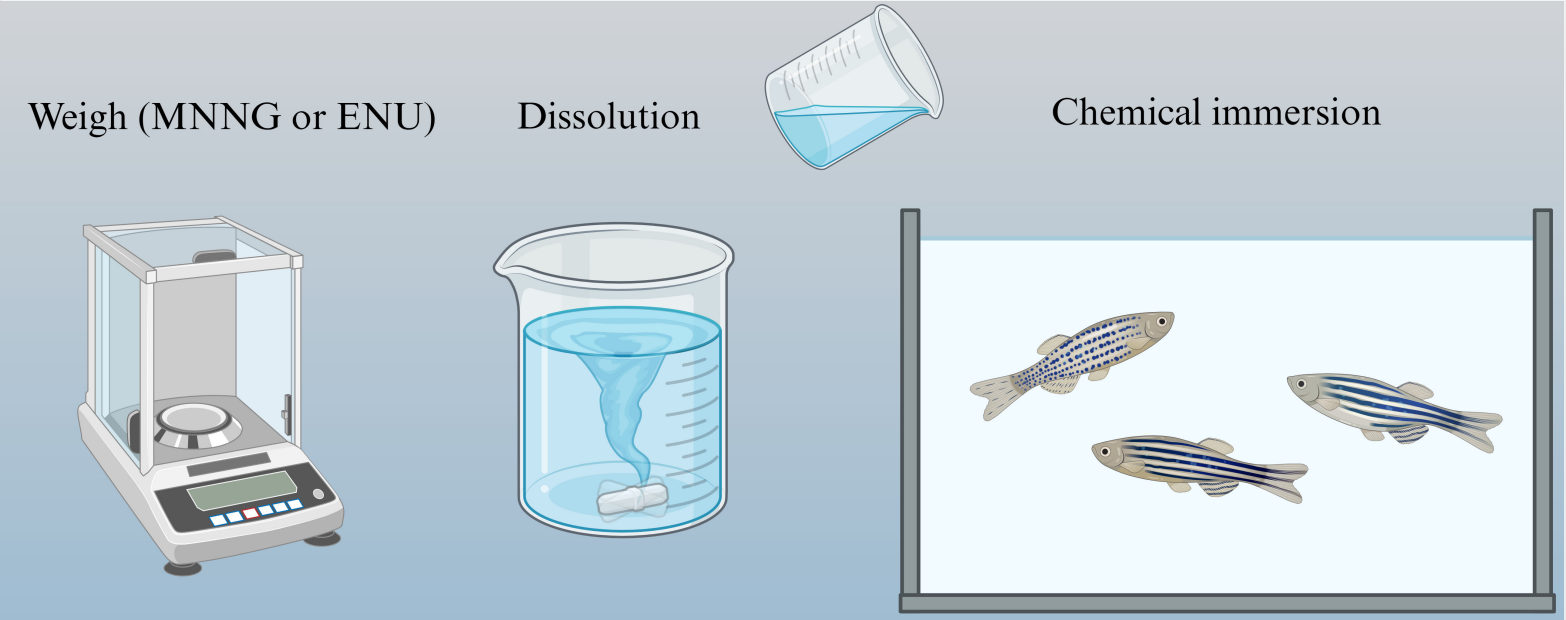
Chemical mutagenesis of zebrafish. Zebrafish were mutated directly with chemical drugs, but the resulting zebrafish was more likely to develop other cancers.

ENU is a well-established alkylating mutagen that primarily induces point mutations through the ethylation of DNA bases. (71) This chemical has been widely used to investigate genetic alterations linked to cancer development, including gliomas. ENU exposure in zebrafish typically occurs in a controlled manner, with optimal concentrations ensuring a balance between mutagenicity and toxicity. Studies suggest that repeated exposure to 3-3.5 mM ENU over 2–4 weeks can effectively induce mutagenesis in zebrafish embryos (72) (**Figure 4**).

For example, Solnica-Krezel et al. exposed male wild-type zebrafish to 3mM ENU for 1 h/day over a pan of 2–4 weeks. (73) After exposure, these males were crossed with wild-type females. The offspring, examined under confocal microscopy, revealed head enlargement and localized masses in the brain vasculature, which are characteristic features of glioma-like tumors. Similarly, Wienholds et al. administered 3.0 mM ENU to four-month-old male zebrafish, repeating the exposure six times. (74) The offspring were subsequently screened for glioma-related markers and tumor phenotypes. Although ENU successfully induces random mutations, its application in glioma modeling is limited by its relatively low efficiency and the non-specific nature of the tumor phenotypes induced, which may affect multiple organs, including the liver and testis, complicating the identification of glioma-specific markers.

#### 3.1.2 MNNG-induced glioma model

MNNG, is another powerful alkylating agent that induces mutations by adding methyl groups to the DNA, leading to the disruption of normal cellular processes and subsequent tumor formation. MNNG has been shown to cause glial cell hyperplasia and abnormal differentiation, facilitating glioma development by activating cellular signaling pathways involved in proliferation and survival. (75).

Several methods have been employed to expose zebrafish to MNNG, including direct immersion, microinjection, and dietary exposure. (76) The direct immersion method involves placing zebrafish embryos or larvae in MNNG solutions, with concentrations of up to 10 ppm for embryos (at 83 hours post-fertilization) and up to 2 ppm for larvae (at three weeks post-hatching). Short-duration exposures to MNNG in these concentrations have been shown to induce glioma-like characteristics, such as head enlargement and abnormal swimming behaviors (**Figure 4**).

Microinjection allows for more localized mutagenesis by directly injecting MNNG into the zebrafish embryos. This technique enhances the likelihood of developing glioma tumors specifically in the brain, providing a more focused model for glioma research. Lastly, dietary exposure involves feeding zebrafish MNNG-treated food for extended periods, up to three months. This method has been shown to induce a range of mesenchymal tumors, including gliomas, hemangiomas, and sarcomas, highlighting the broad mutagenic potential of MNNG.

Screening of zebrafish exposed to MNNG is conducted through confocal laser microscopy, identifying glioma-related phenotypes in embryos and larvae, such as head enlargement and abnormal swimming patterns. Despite its effectiveness in generating glioma models, MNNG induction can result in tumors in multiple organs, including the liver and testis, complicating the identification of glioma-specific phenotypes.

### 3.2 Genetic engineering in zebrafish glioma models

Genetic engineering is one of the important means to create glioma models. There are many ways to create zebrafish glioma models through genetic engineering.

#### 3.2.1 Activation of the EGFR/RAS/ERK/AKT pathway via the Zic enhancer

To explore glioma driven by specific oncogenic pathways, as shown in **Figure 3B**, Marie Mayrhofer et al. utilized the Gal4-UAS binary expression system, which enables targeted expression of oncogene in zebrafish. (77) In this model, the Ethmz5 driver line expresses a codon-optimized Gal4 transcription factor under the control of the zic4 enhancer, which is specifically active in proliferative regions of the developing CNS. By crossing this driver line with zebrafish carrying various oncogenes under the UAS promoter, the researchers were able to activate the EGFR/RAS/ERK/*AKT* pathway, which is critical in glioma pathogenesis. (78) This activation led to tumor-like growths in the zebrafish brain.

Brain imaging of zebrafish at juvenile and adult stages (ranging from 1–14 months) revealed vsignificant malformations, particularly in the telencephalon, fourth ventricle, and diencephalon. Tumor incidence was assessed over time, with glioma-like growths appearing in 36.6% of zebrafish by 6 months and 49% by 9 months of age upon activation of the AKT pathway. These findings underscore the efficacy of the EGFR/RAS/ERK/AKT pathway as a driver of glioma in zebrafish and highlight the suitability of zebrafish models for studying glioma progression in an age-dependent manner.

#### 3.2.2 Modeling glioma through targeted deletion of Nf1, Tp53 and Rb1

Glioma development in humans is frequently associated with mutations in key tumor suppressor genes, including RTK/Ras/PI3K, RB, and *TP53* pathways. (79) To replicate these genetic alterations in zebrafish, Luo et al. employed the CRISPR/Cas9-based gene editing to targeted the *Nf1*, *Tp53*, and *Rb1* genes. (80) Using a modified U6–3 promoter vector, the researchers inserted specific guide RNA (gRNA) sequences to induce precise gene deletions in these critical tumor suppressor genes. A Cas9-T2A-mCherry construct was used to facilitate gene expression under the control of the gfap promoter, which is active in glial cells, ensuring that the genetic modifications occurred specifically in the brain. Embryos were injected with the CRISPR constructs, and successful modifications were confirmed through histological analysis, immunohistochemistry, and confocal microscopy. Zebrafish embryos exhibiting a “curved body” phenotype were identified as candidates for further study, as this phenotype correlated with the development of cerebellar gliomas and associated motor dysfunctions. Histological analysis of the edited zebrafish revealed significant structural disruption in the brain, with gliomas invading the fourth ventricle, similar to human glioma progression. These findings were confirmed by hematoxylin and eosin staining and confocal imaging, which demonstrated the presence of glioma tissues in these regions. This genetic engineering approach not only mimicked the key genetic alterations found in human gliomas but also provided a powerful tool for dissecting the molecular mechanisms of gliomagenesis. The zebrafish model allowed for detailed study of the roles of *Nf1*, *Tp53*, and *Rb1* mutations in glioma formation and progression.

### 3.3 Xenotransplantation in zebrafish glioma models

Xenotransplantation models in zebrafish provide an invaluable tool for studying glioma progression **Figure 3C**, offering the advantage of real-time *in vivo* observation of tumor growth, invasion, and response to therapy. This approach involves transplanting glioma cells into zebrafish embryos or larvae, enabling the detailed tracking of tumor characteristics in a transparent organism. Several glioma cell lines have been used in xenotransplantation studies, with each exhibiting distinct invasion patterns, tumorigenicity, and responses to therapeutic agents. Here, we review the major glioma cell lines used in zebrafish xenograft models: U87, GBM9, and U251MG (**Table 4**).

**Table 4:** Summary of xenotransplanted glioma cells in zebrafish models.

Cell line	Cell Count (Cells)	Invasion extent and mortality	Treatment	References
U87	1000	20% brain invasion, 40% mortality	PI3K inhibitor LY294002	(81)
U251MG	25-100	100 µm invasion, 11% mortality	Ardipusilloside III	(83)
U87-MG vIII	————	Higher invasion, increased mortality compared to U87	Anti-OPN antibody	(85)
GBM9	10-25, >25	40% mortality (low cell count), 100% mortality (high cell count)	SphK inhibitor	(82)
GBM patient-derived cells PDX	50-100	18% mortality	Temozolomide	(86)
GBM stem cell culture	300-800	————	MMP-9 inhibitors	(87)

#### 3.3.1 U87 glioblastoma cells

**Figure 5:**
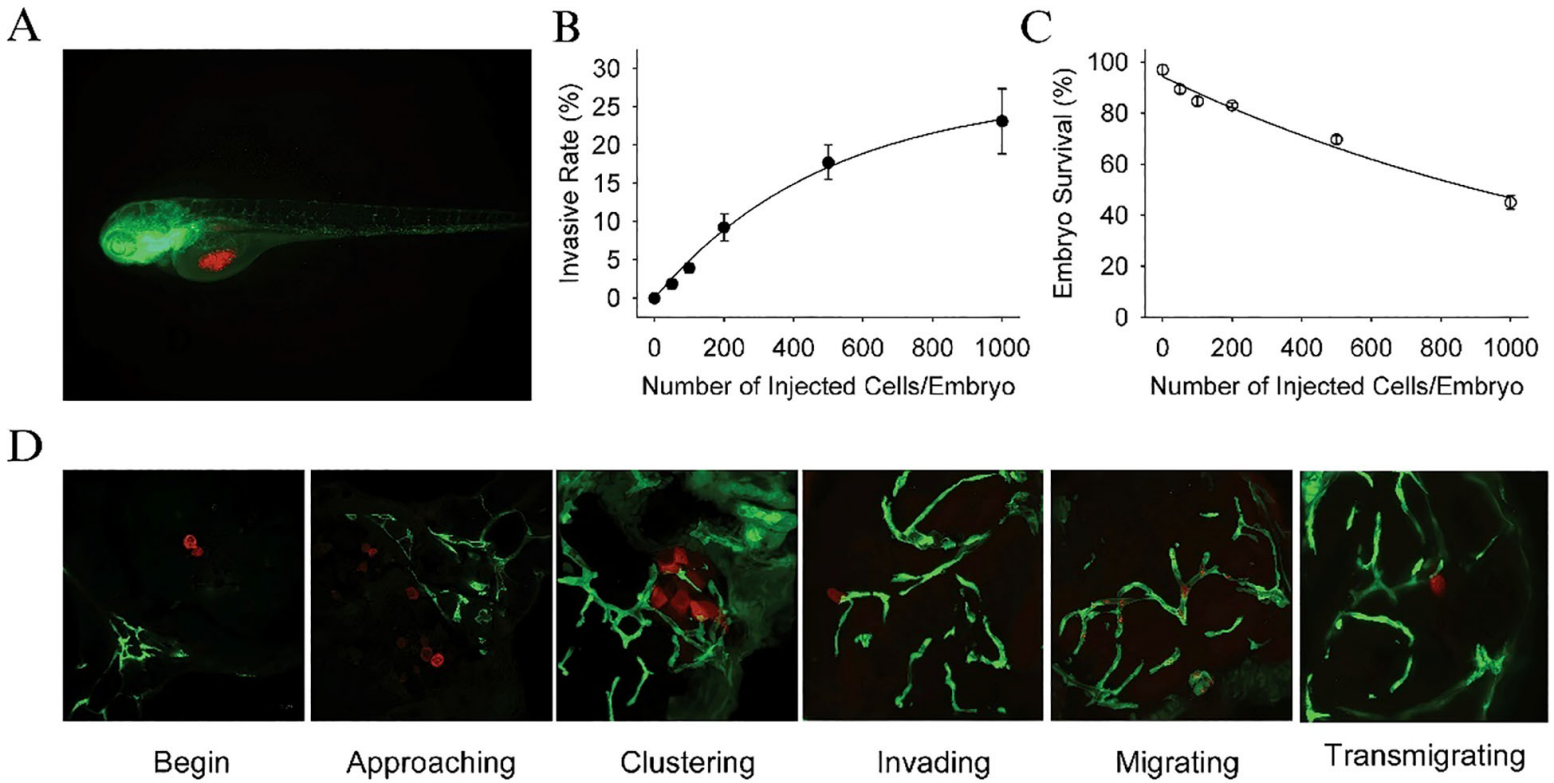
**(A)** Dual color confocal image shows that U87 sphere cells (RFP labeled, red) were microinjected into the middle of yolk *sac* within Tg (*fli1*:EGFP)^y1^ transgenic zebrafish embryos (EGFP labeled, green). **(B)** Different numbers of U87-RFP glioma sphere cells were microinjected into Tg (*fli1*:EGFP)^y1^embryos (n = 300 in each group), and the percentage of embryos with invasive tumor cells was quantitated. **(C)** The survival rate of Tg (*fli1*:EGFP)^y1^zebrafish embryos microinjected with different numbers of U87-RFP glioma sphere cells (n = 300 in each group). **(D)** Representative dual color confocal images of RFP-labeled U87 sphere cells within Tg (*fli1*:EGFP)^y1^zebrafish embryos at the different invasive stages. Red: RFP-labeled U87 sphere cells; Green: Tg (*fli1*:EGFP)^y1^microvessels.

The U87 cell line, one of the most widely used glioblastoma models, is known for its aggressive invasion and growth properties *in vivo*. In a study by Yang et al. (81) U87 cells were transfected with a red fluorescence protein (RFP) plasmid and microinjected into zebrafish embryos at 36 hours post-fertilization. The embryos were cultured at 35°C and monitored for tumor growth using confocal microscopy. The results revealed that U87 cells, once injected, formed secondary tumor nodules, which were typically located around the neurons, blood vessels, and leptomeninges, indicative of glioma’s characteristic pattern of invasion. Furthermore, tumor progression was associated with a significant reduction in embryo survival rates, which decreased to 40% when approximately 1,000 cells were injected. U87 cells were found to occupy nearly 20% of the zebrafish brain, highlighting their invasive nature (**Figure 5**).

#### 3.3.2 GBM9 neurosphere cells

GBM9 cells, derived from patient cortically localized glioblastomas, represent a highly aggressive and clinically relevant glioma model. Welker et al. (82) employed xenotransplantation of both serum-grown adherent GBM9 cells and neurospheres into zebrafish embryos at 36 hours post-fertilization. Confocal imaging over time revealed extensive tumor progression with significant brain infiltration. Notably, zebrafish xenografted with GBM9 cells exhibited abnormal swimming behaviors, including twitching and circling, suggesting impaired brain function. The study showed dose-dependent lethality, with zebrafish injected with over 25 cells exhibiting 100% mortality, while the median survival time decreased significantly with higher cell numbers. These results emphasize the invasiveness and lethal potential of GBM9 cells, making them a valuable tool for modeling aggressive gliomas and evaluating potential therapies.

#### 3.3.3 U251MG glioma cells

**Figure 6:**
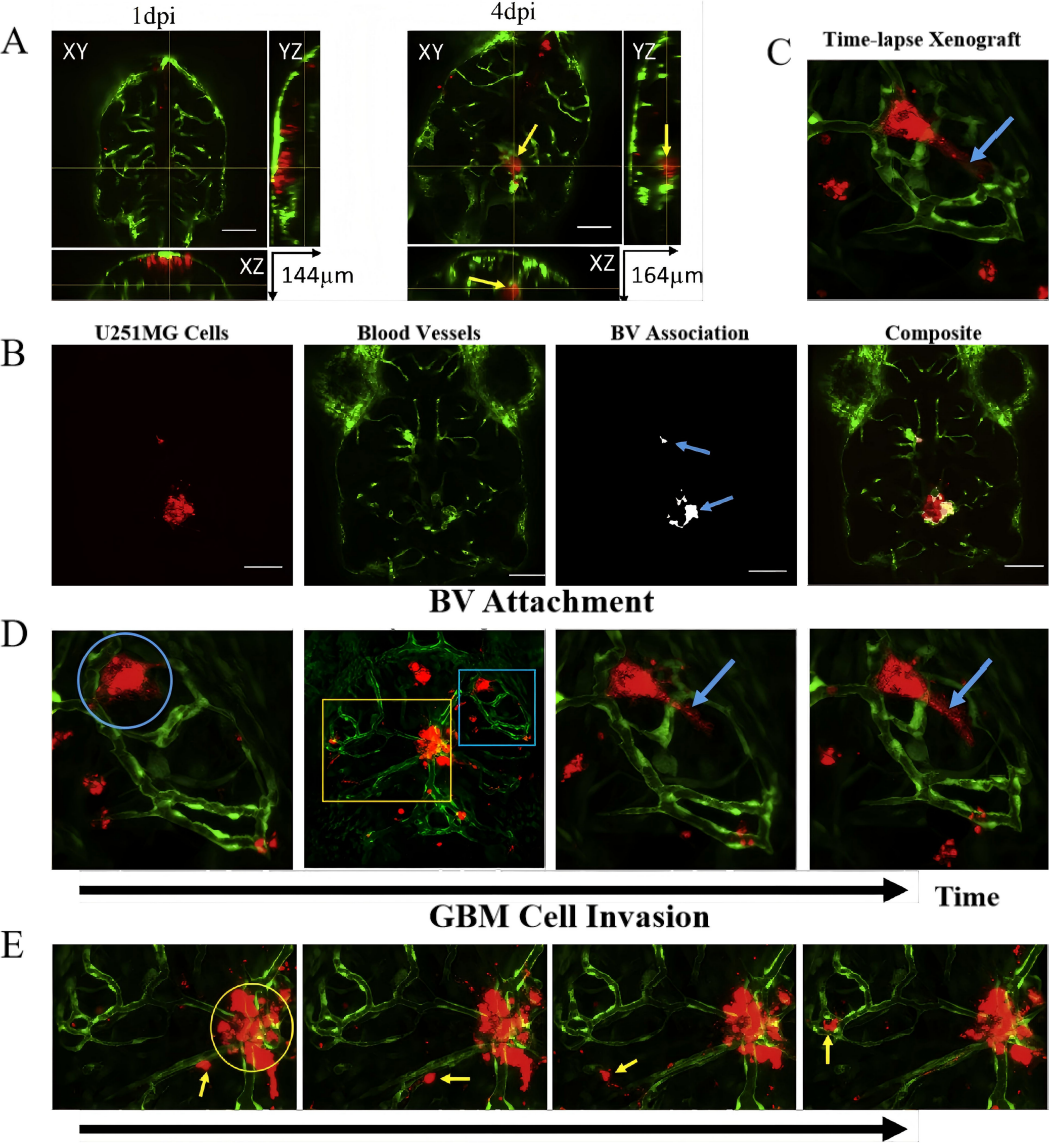
Zebrafish brain xenografts. **(A)** Orthogonal views of zebrafish brain sections showing microtumor formation by U251MG cells (red) and blood vessels (green) at 1 day (left) and 4 days (right) post-injection. yellow arrow indicates tumor formation deep in the brain, surrounded by blood vessels; **(B)** Quantitative analysis of U251MG cell association with blood vessels (BV), showing the microtumors (100 µm scale bar); **(C)** 3D maximum projection frame from a time-lapse video showing blood vessels (green) and transplanted U251MG cells (red) in the zebrafish brain, highlighting regions of tumor invasion (yellow box) and blood vessel (blue box); **(D)** Time-lapse images demonstrating U251MG cells attaching to and retracting from blood vessels (blue circles) with pseudopodia (blue arrows); **(E)** Time-lapse imaging showing U251MG cell invasion (yellow arrow) and non-invasive cells (yellow circles) within the zebrafish brain.

The U251 cell line, derived from human astrocytoma, is frequently used to study glioma progression, particularly in examining tumor-cell interactions with the vasculature. In research by Gamblea et al. (83) U251MG cells were cultured and labeled with CM-dI fluorescent dye before microinjection into the hindbrain ventricle of zebrafish embryos. Tumor progression was monitored using confocal microscopy at 1 and 4 days post-injection, revealing that U251MG cells adhered to the blood vessels within deep brain regions and formed microtumors. The cells also exhibited invasive behavior, with pseudopodia extending toward surrounding brain structures (84). The interactions between U251MG cells and the vasculature were further elucidated using time-lapse imaging, which revealed that U251MG cells integrated with blood vessels and exhibited significant invasion into the zebrafish brain parenchyma, mimicking key features of human glioma progression (**Figure 6**).

While human glioma cell lines such as U87 and U251 have historically provided insights into basic tumor biology, their prolonged *in vitro* culturing has significantly altered key features of glioma, including invasiveness, genomic heterogeneity, and stemness. Given the increasing availability of patient-derived glioma stem-like cells and organoid-based models (88), future studies should prioritize more physiologically relevant systems for both mechanistic research and translational validation.

In general, the chemical mutagenesis zebrafish model is easy to operate and can be screened in large quantities. It is suitable for preliminary exploration of carcinogenic mechanisms. However, its disadvantage is that carcinogenesis is highly random and the tumor type cannot be determined. Gene editing to establish a glioma model can be used through CRISPR/Cas9, TALEN or transgenic strategies to target and regulate key oncogenes or tumor suppressor genes (such as Tp53, IDH1, EGFR, PDGFRA) in zebrafish to induce brain glioma formation. The disadvantage is that the cycle is long and the technical difficulty is high. Xenotransplantation modeling is rapid, and the transparency of zebrafish can be used to observe dynamic processes such as tumor cell migration, invasion, and angiogenesis. In terms of limitations, early embryonic stages are usually used, and there is no mature immune system. In addition, human cells are transplanted, which cannot simulate immune responses. In recent years, the development of TEAZ (Transgene Electroporation in Adult Zebrafish) technology has provided a new approach for the construction of zebrafish tumor models. This method introduces DNA constructs containing specific genes into adult zebrafish through electroporation, achieving spatiotemporal specific induction of tumors. Compared with traditional transgenic methods, TEAZ is easier to operate and is suitable for individuals with intact immunity, providing a powerful tool for studying the occurrence, progression and metastasis of tumors (89).

## 4 Application of zebrafish model in glioma treatment

### 4.1 Contribution of zebrafish models to understanding glioma origins

Zebrafish models have pivotal in enhancing our understanding of glioma’s cellular origins and the molecular mechanisms involved in its initiation. In zebrafish, gliomas preferentially form in the periventricular zone, where neural progenitor cells expressing Ptf1a and Her4 play a crucial role in tumor initiation. This observation was first highlighted by Jung et al. (90) who demonstrated that co-expression of *DARac1* and *DAAkt1* accelerates glioma formation, resulting in more aggressive and invasive tumors. Gene expression profiling revealed upregulation of *survivin2*, *cyclin D1*, *β-catenin*, and *snail1a*, while *E-cadherin* expression was reduced, indicating the involvement of epithelial-to-mesenchymal transition in gliomagenesis. These findings suggest that the transition from an epithelial to a mesenchymal phenotype is critical for glioma progression and may provide a target for future therapeutic strategies.

In addition to the Akt pathway, zebrafish models have shed light on the role of Yes-associated protein (YAP) in glioma. Mayrhofer et al. (91) demonstrated that co-expression of dominant-active *YAP* (*YAPS5A*) and *HRASV12* in zebrafish induced highly invasive tumors, distinguishing malignant gliomas from benign lesions. This underscores the importance of YAP in glioma progression and highlights it as a potential therapeutic target.

Furthermore, the sonic hedgehog (shh) signaling pathway has been implicated in glioma formation. (92) Activation of Shh signaling in zebrafish, driven by Smoa1, resulted in glioma-like tumors in the brain and retina. (93) This observation supports the role of Shh signaling in the regulation of neural progenitor cells and its potential contribution to glioma initiation. In a related study, Ju et al. (94) used the *krt5* gene promoter to drive *SmoA1* expression in neural progenitors, leading to the development of optic pathway gliomas with characteristics of radial glial cells and progenitor populations. These tumors exhibited overexpression of *Mdm2*, a negative regulator of *Tp53*, further linking Shh pathway dysregulation to glioma initiation.

The zebrafish model, therefore, provides a powerful system for dissecting the cellular and molecular events driving gliomagenesis. Key signaling pathways, including Akt, YAP, and Shh, have been shown to play critical roles in glioma formation, offering potential targets for therapeutic intervention.

### 4.2 Mechanistic insights into glioma formation using zebrafish models

The zebrafish models’ transparency, rapid development, and genetic accessibility provide a unique platform for studying tumor initiation, progression, and invasion at cellular and molecular levels. A key development in this area was the creation of an automated, high-throughput transplantation assay for GBM tumors in zebrafish, as demonstrated by Pudelko et al. (95) Their method, which integrates time-lapse and *in vivo* light-sheet microscopy, enables efficient tracking of tumor growth and invasiveness. This approach stands out from traditional models that often require complex and time-consuming intracranial embryo injections. Pudelko’s assay allows the processing of thousands of embryos per hour, positioning zebrafish as a robust vertebrate model for large-scale drug screening and mechanistic studies in glioma research.

Building upon this, Ferrarese et al. (96) enhanced the zebrafish glioma model by incorporating patient-derived glioma cells and employing deep learning techniques for tumor analysis. Their AI-driven system, which utilizes convolutional neural networks, significantly improves data collection efficiency by identifying tumor locations, fish position, and tumor status across imaging frames. This technology allows for continuous monitoring of tumor behavior, such as invasiveness, proliferation, and the impact on host survival, facilitating more comprehensive longitudinal studies.

Moreover, advances in embryo transplantation techniques have allowed researchers to transplant human GBM cultures into blastocyst-stage (3.5 hpf) zebrafish embryos. This model has shown that over 70% of injected embryos develop CNS tumors within 24 hours, with time-lapse confocal microscopy revealing the migration and localization of GBM cells to the neuroprimary zone. This region, essential for neural development, becomes the site of early glioma formation before neuron differentiation is complete. These observations provide valuable insights into the initial stages of glioma cell integration into the host CNS, shedding light on the early tumor-host interactions and cellular migration dynamics that occur during gliomagenesis. (97) The zebrafish model, thus, serves as an invaluable tool in unraveling the complex mechanisms of glioma formation.

### 4.3 Zebrafish models in TME studies and therapeutic screening

Glioma progression is intricately influenced by the TME, a dynamic ecosystem comprising both tumor and non-tumor elements. These components, including cellular and soluble factors, interact with transformed cells to modulate tumor growth, invasiveness, and resistance to therapies. (98) Zebrafish models, with their transparency and ability to model complex biological processes in real-time, have become invaluable for investigating TME dynamics. They allow researchers to study intercellular signaling, including the roles of extracellular vesicles, cytokines, and other mediators, providing new insights into glioma as an interconnected biological network (99).

#### 4.3.1 Angiogenesis and inflammation in glioma progression

Angiogenesis, the formation of new blood vessels, is a hallmark of glioma and a crucial factor in tumor growth, ensuring an adequate supply of nutrients and oxygen to rapidly proliferating cancer cells. Notably, TGF-β1 was shown to significantly enhance angiogenesis, while JNK pathway inhibition markedly reduced vessel formation, highlighting JNK as a potential target for limiting tumor vascularization. In contrast, inhibitors of p38 MAPK, ERK, and PI3K pathways did not impact angiogenesis, underlining the specificity of the JNK signaling pathway in glioma vascular responses.

Further extending these findings, Umans et al. (100) utilized advanced imaging techniques in zebrafish to model perivascular GBM invasion. Their study quantified changes in blood vessel volume and glut1 signal intensity during tumor growth. By observing glioma cells’ interaction with tumor-associated and non-tumor-associated blood vessels, they identified critical tumor-vascular interactions that support glioma cell invasion. This research provides valuable insights into how glioma cells manipulate the vascular niche to facilitate tumor progression and invasion, pinpointing novel therapeutic targets aimed at disrupting these interactions.

In recent years, the zebrafish model has not only made progress in the growth and invasion mechanisms of gliomas, but has also been gradually used to analyze the tumor-related immune microenvironment. Mai Nguyen-Chi et al. (101) used the zebrafish *in situ* transplantation model to observe tumor-induced macrophage aggregation and M1/M2 polarization dynamics, revealing the key role of the innate immune system in the early progression of tumors; Zhang et al. (102) combined transgenic fluorescent labeling technology to track the regulatory mechanism of glioma cells on neutrophil chemotaxis in real time. These results show that zebrafish not only have imaging and intervention advantages, but can also be used to establish a preliminary screening platform for microenvironment intervention targets.

#### 4.3.2 Drug screening and therapeutic evaluation

Zebrafish models are increasingly used for high-throughput drug screening, enabling rapid evaluation of therapeutic efficacy and safety *in vivo*. He et al. (103) demonstrated the potential of zebrafish for testing combination therapies by investigating the effects of ionizing radiation and temozolomide in U251 glioma cell xenografts. Their results showed a significant reduction in tumor size with combined treatment, which was further enhanced by pre-treatment with temozolomide, without notable toxicity to embryonic development. These findings underscores the utility of zebrafish models in optimizing glioma treatment regimens by balancing therapeutic efficacy with safety.

Similarly, Li et al. (104) compared conventional radiotherapy with pulsed low-dose rate radiation using zebrafish glioma models. They found that pulsed radiation more effectively controlled glioma growth while minimizing damage to normal tissues, suggesting it could be a promising alternative to traditional radiotherapy. This highlights the zebrafish model’s capacity to simulate and evaluate treatment responses, facilitating the development of more refined and targeted radiotherapeutic strategies for glioma.

#### 4.3.3 Innovative drug delivery systems in glioma therapy

Overcoming the BBB remains one of the most significant challenges in glioma therapy. Zebrafish models have been instrumental in exploring innovative drug delivery systems designed to address this issue. Jia et al. (105) developed a novel approach using neutrophil-derived exosomes loaded with doxorubicin hydrochloride (DOX) to target gliomas. Exosomes, known for their ability to cross the BBB and their excellent biocompatibility, (106) effectively delivered DOX to brain tumors in zebrafish models. This method reduced systemic toxicity and improved therapeutic outcomes, demonstrating the potential of exosome-based drug delivery as a minimally invasive strategy for treating gliomas.

Additionally, the effects of dl-nordihydroguaiaretic acid (Nordy) were explored in zebrafish glioma models by researchers investigating its impact on GSCs. (107) Nordy was found to inhibit GSCs proliferation and promote their differentiation into astrocyte-like cells, reducing tumor invasiveness and angiogenesis that targeting GSCs through differentiation therapies could offer a novel approach for managing aggressive gliomas, complementing existing treatments by addressing the tumor’s stem cell compartment (108).

#### 4.3.4 Microbiota–immune interactions in glioma

The latest studies have shown that the intestinal microbiota can indirectly affect the immune microenvironment of brain tumors, especially gliomas, by regulating systemic immune responses. (109) Microbial metabolites and the cytokine network mediated by them have been found to regulate the polarization state of tumor-associated macrophages, the infiltration ability of T cells, and the local immunosuppression state of tumors. Although the research on the gut-brain-immune axis in the field of glioma is still in its early stages, this direction can provide new targets for immunotherapy. Zebrafish have potential in studying host-microbe interactions, and their innate immune system is highly conserved with humans. In the future, introducing microbial manipulation technologies (such as germ-free zebrafish and colonization models) into zebrafish glioma models is expected to further clarify how microbial signals shape the immune landscape of glioma.

Through these studies, zebrafish models have proven to be powerful tools for both understanding the complex TME and for therapeutic screening. They provide unique opportunities to study glioma progression in real-time, evaluate the efficacy of novel treatment regimens, and explore cutting-edge drug delivery methods, all while reducing the need for more complex and costly mammalian models.

## 5 Challenges and prospects

**Figure 7:**
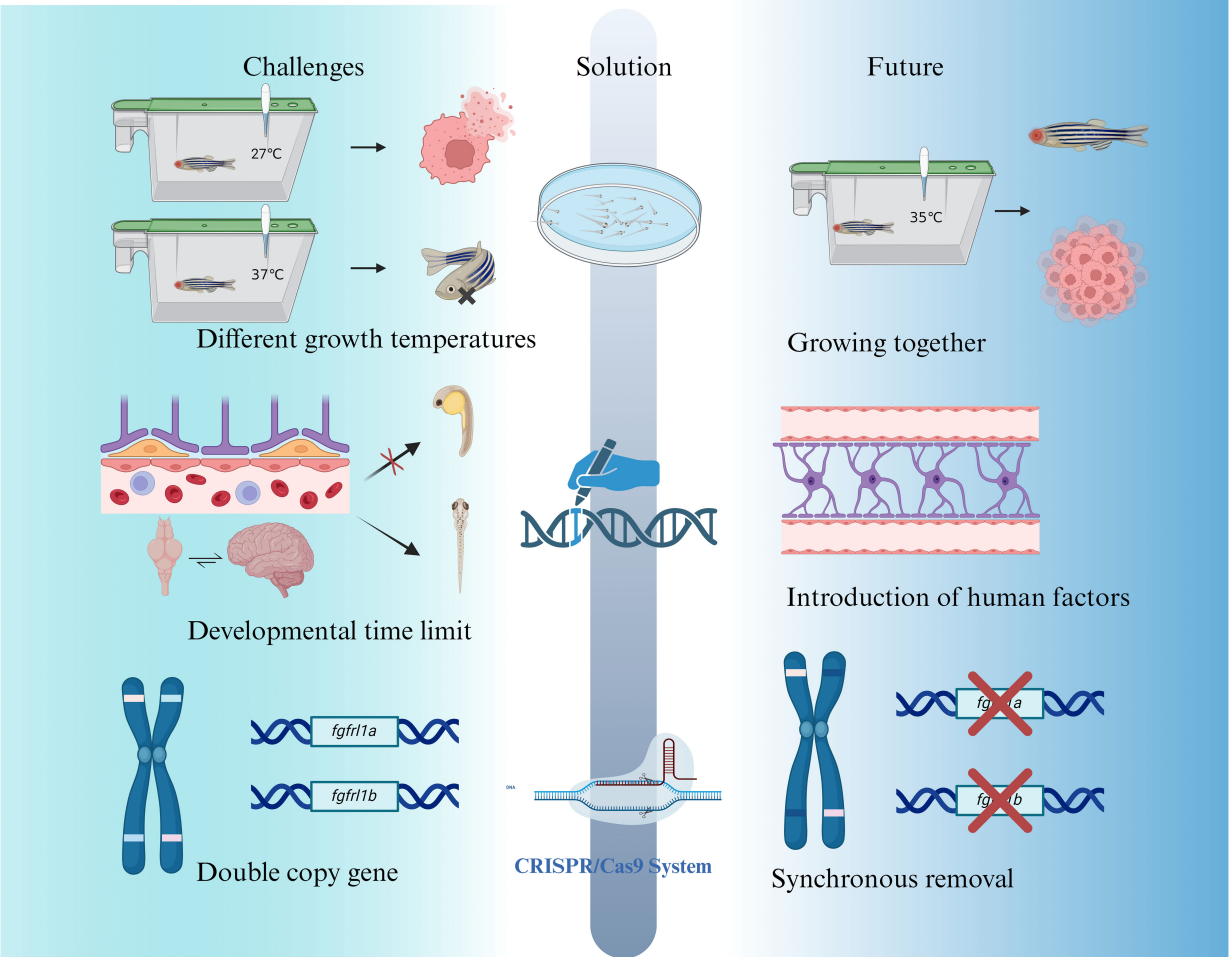
Current challenges, improvement strategies, and prospects for zebrafish as a glioma disease model.

The zebrafish glioma model has emerged as a transformative tool for understanding glioma biology and advancing drug discovery. However, certain limitations restrict its broader applicability, necessitating continued innovation to enhance its translational potential. Below, the key challenges and prospective solutions to address these limitations are outlined (**Figure 7**).

### 5.1 Temperature discrepancies between zebrafish and glioma cells

Zebrafish, as a tropical organisms, thrive at 27-28°C, (110) while glioma cells proliferate optimally at 37°C. (111) Maintaining zebrafish at 28°C impairs glioma cell growth, whereas increasing the temperature to 37°C induces significant stress in zebrafish, resulting in approximately 20% mortality. The current compromise of culturing zebrafish at approximately 35°C mitigates some issues but remains suboptimal, leading to reduced glioma growth rates and affecting experimental reliability. (112) The lower living temperature of zebrafish (about 28–33°C) will affect the expression and function of some temperature-sensitive immune factors, such as the activation of the IFN-γ signaling pathway. To a certain extent, it will weaken the pro-inflammatory response or affect the efficacy evaluation of immune-related drugs.

Future solutions may involve genetic modifications to enhance zebrafish thermal tolerance or metabolism flexibility. Transgenic zebrafish lines capable of thriving at higher temperatures suitable for glioma cell growth could bridge this gap. Additionally, advances in metabolic engineering thermal adaptation research might yield zebrafish mutants optimized for experiments involving human-derived tumor cells.

### 5.2 Developmental and physiological constraints

The immature state of zebrafish embryos presents several limitations for glioma research. For example, CNS in zebrafish embryos lacks myelination until 4–7 dpf, potentially affecting glioma invasion mechanisms reliant on interactions with myelinated axons. Moreover, the absence of a fully formed BBB during early development impacts the accuracy of drug screening and therapeutic testing. In glioma immune research, a key limitation of the zebrafish model is the lack of a mature adaptive immune system in the embryonic and larval stages, which leads to inadequate simulation of T cell-mediated immune responses or immune checkpoint mechanisms. For example, there is currently no way to study the complete mechanism of action of immune checkpoint inhibitors such as PD-1/PD-L1 in zebrafish younger than 3–4 weeks of age.

While the adult zebrafish BBB eventually recapitulates mammalian structure, this embryonic delay challenges its use in preclinical testing of glioma therapies. To address these developmental limitations, researchers are exploring transgenic zebrafish lines engineered to express human-specific cytokines and immune components. (86) Such innovations aim to better mimic the human TME and immune responses. Furthermore, advanced gene-editing technologies, such as CRISPR/Cas9, may be utilized to introduce human-specific markers, improving the model’s physiological relevance to human glioma biology.

### 5.3 Gene duplication and genetic complexity

Unlike mammals, which typically have single copies of genes, zebrafish often possess duplicate genes, adding complexity to genetic manipulation. (113) For example, zebrafish have two copies of the *FGFRL1* gene (*fgfrl1a* and *fgfrl1b*), both located on chromosome 14 with distinct but overlapping functions in tissue expression. (114) This redundancy complicates genetic manipulation, particularly when studying tumor suppressor genes or oncogenes, as single-gene knockouts may not produce the desired phenotypes if compensation occurs via the paralogous gene.

Overcoming this challenge requires the development of precise gene-editing strategies capable of targeting both gene copies simultaneously. CRISPR/Cas9 technologies, combined with multiplexed gRNA designs, offer a promising solution for generating zebrafish glioma models with high genetic fidelity. Such approaches are critical for dissecting the contributions of specific genetic alterations to glioma initiation and progression. In the future, the application of zebrafish in glioma modeling will benefit significantly from the development of a variety of emerging technologies. For example, single-cell transcriptome sequencing (scRNA-seq) can achieve high-resolution analysis of internal heterogeneity and immune cell dynamics in zebrafish brain tumors. CRISPR technology is not only used for targeted editing of tumor-related genes, but can also be applied to precise intervention of immune regulatory pathways, such as macrophage polarization and T cell chemotaxis. In addition, the development of humanized zebrafish models, such as the introduction of human hematopoietic system or MHC molecule expression, is expected to overcome the current limitations in simulating the immune microenvironment. These advances will enhance the translational potential of zebrafish models in immuno-oncology.

## 6 Conclusion

Zebrafish models have transformed glioma research by offering a cost-effective, genetically tractable platform for studying tumor biology, identifying molecular pathways like RTK/Ras/PI3K, RB, and TP53, and conducting high-throughput drug screening. Their transparency and genetic homology with humans enable detailed investigation of glioma pathophysiology and therapeutic responses. While challenges such as temperature constraints, delayed BBB development, and genetic redundancy remain, advances in genetic engineering, humanized lines, and innovative imaging technologies are bridging these gaps. As these models evolve, they promise to accelerate the discovery of novel glioma treatments, paving the way for improved patient outcomes.
