# Supplementary material for: Zebrafish models in glioma research: advances in methodologies, mechanistic insights, and therapeutic frontiers
Source: Front Immunol. 2025 Jun 24;16:1601656. doi: 10.3389/fimmu.2025.1601656 (PMC12234541; doi:10.3389/fimmu.2025.1601656)
Supplement: Supplementary file 1 [file Table1.docx]

***Supplementary Material***

**Zebrafish Models in Glioma Research: Advances in Methodologies, Mechanistic Insights, and Therapeutic Frontiers**

**Runchao Tao ^1†^,Junying Qu ^1†^, Jing Zhao ^1†^, Baihui Wu ^1^, Huaibiao Xu ^1^, Liangwei Yang ^2^, Hongru Qin ^2^, Rongbing Chen ^3^, Qinsi Yang ^4^, Yongwei Cheng ^5^, Wei Wu ^6, 7*^, Da Sun ^1, 7*^, Min Cao ^8*^**

1. ^Institute of Life Sciences & Biomedical Collaborative Innovation Center of Zhejiang Province, Wenzhou University, Wenzhou 325035, China^
2. ^The First School of Medicine, School of Information and Engineering, Wenzhou Medical University, Wenzhou 325000, China^
3. ^Department of Biomedical Engineering, City University of Hong Kong, Hong Kong SAR 999077, China^
4. ^Wenzhou Institute, University of Chinese Academy of Sciences, Wenzhou 325000, China^
5. ^National Engineering Research Center of Cell Growth Factor Drugs and Protein Biologics, Wenzhou Medical University, Wenzhou 325000, China^
6. ^Key Laboratory for Biorheological Science and Technology of Ministry of Education, State and Local Joint Engineering Laboratory for Vascular Implants, Bioengineering College of Chongqing University, Chongqing 400044, China^
7. ^JinFeng Laboratory, Chongqing 401329, China^
8. ^The Quzhou Affiliated Hospital of Wenzhou Medical University, Quzhou People’s Hospital, Quzhou 324000, China^

^* Correspondence: Wei Wu, david2015@cqu.edu.cn; Da Sun, sunday@wzu.edu.cn; Min Cao, mcao@wmu.edu.cn^

^† These authors contributed equally to this work.^

**Table S1**. Key glioma regulatory pathways

| **Gene/Pathway** | **Mutations/Alterations** | **Functional Impact** | **Clinical Relevance** | **References** |
| --- | --- | --- | --- | --- |
| ***TP53*** | Mutations in *TP53* gene | Loss of *p53* function, evasion of apoptosis, uncontrolled proliferation | Poor prognosis, resistance to therapy, glioma recurrence | (K.Sabapathy et al 2019) |
| ***NF1*** | Mutations in *NF1* gene | Loss of *NF1* function, mesenchymal transformation, enhanced invasiveness | Aggressive phenotype, therapy resistance | (S.Perreault et al 2019) |
| **AKT** | Hyperactivation of AKT pathway | Promotes survival, migration, and proliferation of glioma cells | Tumor growth, metastasis, potential therapeutic target | (H.-B et al 2020) |
| **Notch** | Dysregulation (e.g., Notch1) | Regulation of glioma stem cells, self-renewal, invasiveness | Target for glioma stem cell therapies, tumor recurrence | (H. Wang et al 2015) |

**References**

Sabapathy K, Lane DP. Understanding p53 functions through p53 antibodies. *Journal of Molecular Cell Biology*. 2019;11(4):317-329.

Perreault S, Larouche V, Tabori U, et al. A phase 2 study of trametinib for patients with pediatric glioma or plexiform neurofibroma with refractory tumor and activation of the MAPK/ERK pathway: TRAM-01. *BMC Cancer*. 2019;19(1):1250.

Zu HB, Liu XY, Yao K. DHCR24 overexpression modulates microglia polarization and inflammatory response via Akt/GSK3β signaling in Aβ25-35 treated BV-2 cells. *Life Sci*. 2020;260:118470.

Wang H, Zang C, Liu XS, Aster JC. The role of Notch receptors in transcriptional regulation. *J Cell Physiol*. 2015;230(5):982-988.
